# Assessing Rapid Relaxed-Clock Methods for Phylogenomic Dating

**DOI:** 10.1093/gbe/evab251

**Published:** 2021-11-09

**Authors:** Jose Barba-Montoya, Qiqing Tao, Sudhir Kumar

**Affiliations:** 1 Institute for Genomics and Evolutionary Medicine, Temple University, Philadelphia, Pennsylvania, USA; 2 Department of Biology, Temple University, Philadelphia, Pennsylvania, USA; 3 Center of Excellence in Genomic Medicine Research, King Abdulaziz University, Jeddah, Saudi Arabia

**Keywords:** relaxed molecular clock dating, phylogenomics, relative rate framework, penalized likelihood, least-squares

## Abstract

Rapid relaxed-clock dating methods are frequently applied to analyze phylogenomic data sets containing hundreds to thousands of sequences because of their accuracy and computational efficiency. However, the relative performance of different rapid dating methods is yet to be compared on the same data sets, and, thus, the power and pitfalls of selecting among these approaches remain unclear. We compared the accuracy, bias, and coverage probabilities of RelTime, treePL, and least-squares dating time estimates by applying them to analyze computer-simulated data sets in which evolutionary rates varied extensively among branches in the phylogeny. RelTime estimates were consistently more accurate than the other two, particularly when evolutionary rates were autocorrelated or shifted convergently among lineages. The 95% confidence intervals (CIs) around RelTime dates showed appropriate coverage probabilities (95% on average), but other methods produced rather low coverage probabilities because of overly narrow CIs of time estimates. Overall, RelTime appears to be a more efficient method for estimating divergence times for large phylogenies.

Significance

Many studies now report using RelTime, treePL, and least-squares dating (LSD) approaches to analyze phylogenomic data sets that usually contain hundreds or thousands of sequences and genes. However, the performance of these methods has not been thoroughly compared. Therefore, researchers need a comparative assessment of rapid methods to determine the advantages of preferring one approach over another in certain evolutionary situations. This article presents accuracy, bias, and coverage probabilities of time estimates produced by applying rapid methods to computer-simulated data sets in which evolutionary rates varied extensively throughout the phylogeny. The RelTime method produced estimates with higher accuracy, lower bias, and confidence intervals suitable for biological hypothesis testing.

## Introduction

Phylogenomic data sets encompassing many species and genes are now routinely assembled for building molecular timetrees, thanks to many advances in sequencing technologies (dos Reis et al. 2016; Tao, Tamura, and Kumar 2020). These data sets can be challenging to analyze using Bayesian dating methods because of their large calculation time and high computer memory requirements. So, many rapid non-Bayesian molecular dating methods are used to infer divergence times of data sets containing hundreds and thousands of sequences and lineages (reviewed in Tao, Tamura, and Kumar 2020). Both rapid and Bayesian methods allow branch evolutionary rates to be heterogeneous across the phylogeny (Sanderson 1997, 2002; Tamura et al. 2012, 2018; Tao, Tamura, and Kumar 2020). Computational requirements of rapid methods are a small fraction of that required by Bayesian methods (dos Reis et al. 2016; Tao, Tamura, and Kumar 2020). Two fast relaxed-clock methods are being applied more often than all others. One is the RelTime method based on the relative rate framework (RRF) (Tamura et al. 2012, 2018) as implemented in MEGA (Tamura et al. 2021). The other is the penalized likelihood (PL) method, first implemented in r8s (Sanderson 2002, 2003) and now available in other software, including treePL (Smith and O’Meara 2012) and chronopl/chronos (ape R package, Paradis 2013). RelTime and treePL have been used for node dating in hundreds of research articles in the last five years.

In brief, the PL approach estimates divergence times using an optimization algorithm that minimizes (smooths) the squared differences between ancestral and descendant branch rates in a phylogenetic tree (Sanderson 1997, 2002). PL applies a penalty to rate changes between adjacent branches while maximizing the likelihood of the data, thus allowing estimation of both rates and times. Implementation of this method in treePL includes a global smoothing parameter estimated through a cross-validation procedure, which controls the magnitude of penalizing rate changes relative to the likelihood (Smith and O’Meara 2012). The penalized likelihood score is then maximized, allowing the estimation of branch-specific rates and divergence times across the phylogenetic tree (Sanderson 2002). The CIs of treePL time estimates, which represent the uncertainty of estimates, are often obtained via a bootstrap procedure (Sanderson 1997, 2003; Thorne and Kishino 2005; Yang 2014).

In contrast, the RelTime approach is based on RRF, which also relaxes the molecular clock but does not require a global parameter for rate smoothing. Instead, RRF solved a system of equations providing relationships of ratios of lineage rates and lengths for every node in the phylogeny (Tamura et al. 2018). In RRF, an evolutionary lineage encompasses the stem branch and the clade originating from it, which is different from PL in which branch rates are considered. Ultimately, RRF produces analytical formulas for directly estimating lineage rates and node times as a function of branch lengths in the phylogeny. This analytical formulation has been utilized to derive equations to estimate CIs (no bootstrapping) for node times in a way that incorporates both rate heterogeneity among lineages and errors in branch length estimates (Tao, Tamura, Mello, et al. 2020).

Many studies have tested the RelTime method and compared its accuracy with Bayesian methods. These studies applied RelTime in the analysis of empirical data sets as well as data sets simulated under a variety of evolutionary scenarios for large data sets (Tamura et al. 2012, 2018; Mello et al. 2017; Battistuzzi et al. 2018; Barba-Montoya et al. 2020; Beavan et al. 2020; Tao, Tamura, and Kumar 2020). They report comparable performance of RelTime and Bayesian methods, with and without applying internal calibrations. In contrast, testing of treePL’s performance for large data sets has been somewhat limited. For example, Smith and O’Meara (2012) suggested that treePL estimated times were overall as accurate as of its predecessor r8s (Sanderson 2003) and that it performed better than PATHd8, which assumes a molecular clock (Britton et al. 2007). However, r8s implementation was tested in Tamura et al. (2012) and found not to perform as well as RelTime in some situations. Other evaluations of PL methods have been made for phylogenies with a small number of sequences (Sanderson 1997; Ho et al. 2005).

Some studies have reported similar performance between treePL and Bayesian methods, but they used internal calibrations, making it difficult to assess the relative power of the time structure imposed by calibrations versus that inferred by the PL approach (Smith and O’Meara, 2012; Gunter et al. 2016; Carruthers et al. 2020). Moreover, the performance of treePL using phylogenies in which branches evolved with uncorrelated rates has not been evaluated. Furthermore, there is a need to assess the accuracy of treePL’s divergence time estimates node-by-node and their CIs’ coverage probabilities (CPs).

Importantly, the performance of RelTime and treePL has not been compared thoroughly on the same data sets or under a variety of evolutionary rate heterogeneity models. Researchers need such comparisons to determine if there are advantages to preferring a method in certain evolutionary situations. Therefore, we have compared the accuracy of both RelTime and treePL by analyzing data sets generated by computer simulations in which branch evolutionary rates varied greatly following autocorrelated and uncorrelated rate models. We focused our investigation on dating analyses in which no time constraints were used, except for a single root calibration. This choice allowed us to directly examine the power of PL and RRF frameworks in dealing with rate variation without the aid of internal calibrations (see the Discussion).

In addition to evaluating the accuracy and bias, we have compared CPs of the CIs produced by RelTime and treePL. This is important because the bootstrap approach to estimate CIs around treePL estimates can only account for errors in branch length estimates, which ignores variances caused by the heterogeneity of rates among lineages (Thorne and Kishino 2005; Yang 2014). This contrasts with the analytical method available for RelTime that accounts for variances contributed by errors associated with branch length estimation and rate variation among branches (Tao, Tamura, Mello, et al. 2020).

In addition to treePL and RelTime, we have included an evaluation of an LSD approach (To et al. 2016) because its statistical basis is different from the RRF and PL approaches. The LSD method assumes the noise in molecular rates to be approximately normally distributed and independent among branches. In LSD, CIs are calculated by resampling the branch lengths of the estimated timetrees to build a set of trees for CI inference; it uses a Poisson distribution to generate resampled branch lengths.

In the following, we present results from our comparison of the accuracy, bias, and coverage probabilities of time estimates produced by applying these three rapid methods to computer-simulated data sets in which evolutionary rates varied extensively throughout the phylogeny.

## Results

### Impact of Rate Variation Process on Time Estimates

**Fig. 1.**
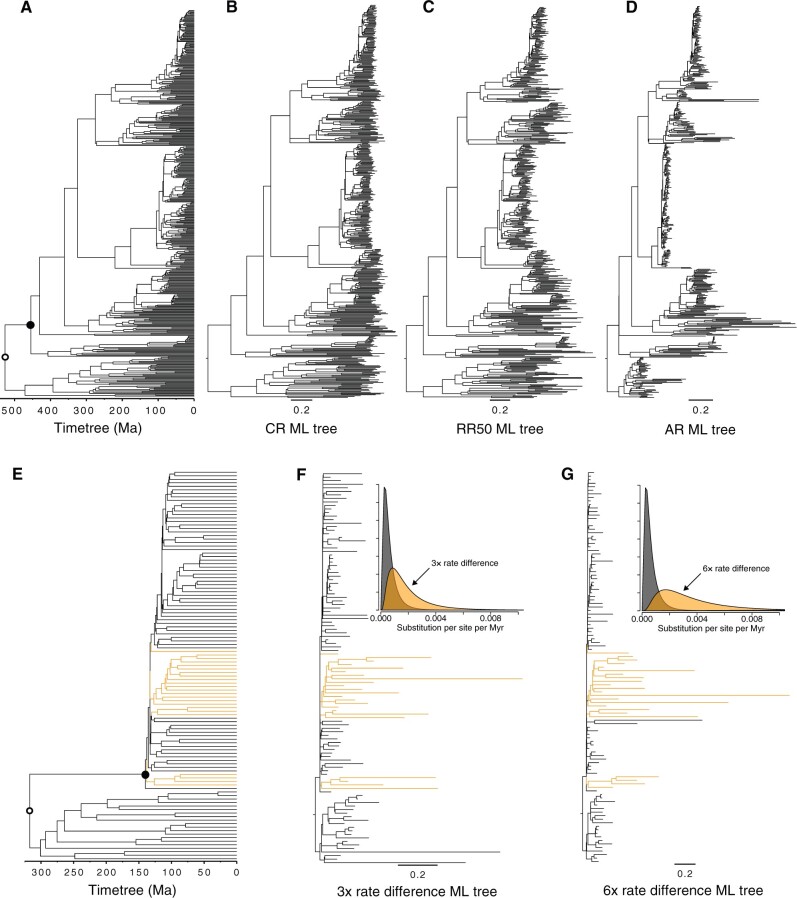
Phylogenies used in this study. Phylogeny of 446 taxa showing calibrated nodes (*A*), the tree has been scaled to time based on the time estimates from the Timetree of Life (Hedges and Kumar, 2009). Calibrations are represented for two nodes. 1) A uniform distribution U(min, max) for the rooting ingroup calibration U(444.6, 464.6 Ma) was applied in both RelTime and treePL analyses. For LSD, the calibration was fixed to 454 Ma (closed black dot). 2) In treePL analysis, a max constraint (527 Ma) was applied on the root (open black dot). In RelTime and LSD, the rooting outgroup was excluded from the analysis. Simulated ML phylogenies with branch lengths for CR (*B*), RR50 (*C*), and AR (*D*) replicates. Note that branch lengths from RR50 (*C*) and AR (*D*) ML phylogenies display a large rate variation. Phylogeny of 111 taxa showing calibrated nodes (*E*), the tree has been scaled to time based on the time estimates from Beaulieu et al. (2015). Calibrations are represented for two nodes. 1) A uniform distribution for the rooting ingroup calibration U(135, 145 Ma) was applied in both RelTime and treePL analyses. For LSD, the calibration was fixed to 140 Ma (closed black dot). 2) In the treePL analysis, a max constraint (317 Ma) was applied on the root (open black dot). In RelTime and LSD, the rooting outgroup was excluded from the analysis. Simulated ML phylogenies with branch lengths for (*F*) 3× and (*G*) 6× rate difference replicates. Faster molecular rates were simulated by independently drawing rates for the orange branches from a lognormal distribution with a mean 3× and 6× higher (orange curves) than all other branches in the tree (gray curves) (*F* and *G*).

We quantified the accuracy and bias of time estimates produced by RelTime, treePL, and LSD for data sets in which sequences were evolved with constant and variable evolutionary rates. We used data sets previously simulated by Tamura et al. (2012). Their model timetree consisted of 446 species derived from the bony-vertebrate clade in the Timetree of Life (Hedges and Kumar 2009) (fig. 1*A*). One hundred sequence alignments were generated under molecular clock as a baseline (CR data sets; fig. 1*B*). Another 100 alignments were generated by varying rates independently on each branch by ±50% of the overall rate (RR50 data sets; fig. 1*C*). And, finally, 100 data sets were selected in which the rate variation was autocorrelated (AR data sets; fig. 1*D*).

We also used data sets previously simulated by Beaulieu et al. (2015) for a model timetree of 111 taxa (fig. 1*E*) derived from the analysis of the age of land plant clades. Assuming autocorrelated rates among lineages, Beaulieu et al. (2015) simulated scenarios in which four clades in the phylogeny evolved three times higher rate than the remaining taxa (3× data sets; fig. 1*F*). They also simulated a more extreme six times rate difference scenario (6× data sets; fig. 1*G*). The simulation system and model parameters used are presented in the Materials and Methods.

#### Accuracy of Individual Node Times

**Fig. 2.**
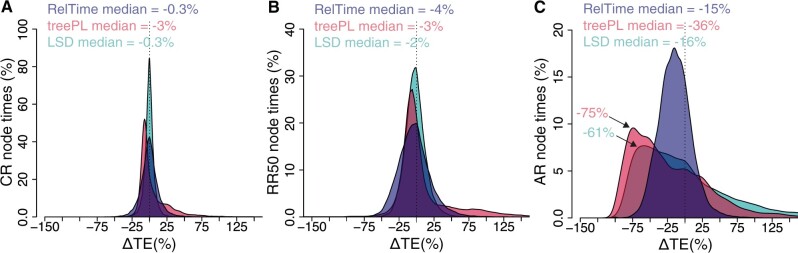
Distributions of the differences between estimated and true node times (ΔTEs) for CR (*A*), RR50 (*B*), and AR (*C*) data sets for times inferred by RelTime (blue curve) treePL (red curve) and LSD (green curve). The distribution in *A–C* was truncated for visual clarity, which excluded a few outliers. The median of each distribution is shown and the arrows mark modes of treePL and LSD distributions for the AR data set in panel *C*. The standard deviations for RelTime, treePL, and LSD, respectively, are as follows in panel (*A*) 13, 24, 7; (*B*) 23, 112, 15; and (*C*) 23, 57, 82.

We first present results for data sets simulated using the model tree in figure 1*A*. When the evolutionary rates were constant (CR), the distribution of differences between RelTime estimated times and true times (ΔTEs) was centered close to zero, with a median equal to –0.3% (fig. 2*A*). The distribution around zero seems symmetrical, but some recent node times were estimated with larger errors.

The distribution of treePL ΔTEs was neither symmetrical nor centered close to zero (fig. 2*A*). Many node times were underestimated, resulting in an overall tendency to underestimate times (median ΔTE = –3%; fig. 2*A*). That is, treePL was sensitive to even small amounts of rate deviation from a strict molecular clock caused by the stochastic nature of the evolutionary process. In contrast, LSD ΔTE distribution was centered on zero (fig. 2*A*).

When evolutionary rates varied independently from branch to branch (RR50 data sets), the performance of treePL deteriorated. The tail of the ΔTE distribution became longer (fig. 2*B*). In RelTime and LSD, the distribution of ΔTEs was still symmetrical, although slightly off-center (fig. 2*B*). Dispersions of ΔTEs for RR50 data sets were greater than CR data sets because of greater rate variation among lineages in the RR50 phylogenies (compare fig. 1*B* and *C*).

The performance of treePL for AR data sets was much worse than RR50 data sets, as the median underestimate grew to –36% (fig. 2*C*). Overall, the acute rate variation created by the autocorrelation of branch rates made it more challenging to estimate dates (compare fig. 1*C* and *D*). But the effect on treePL and LSD performance was much more severe. RelTime ΔTE distribution maintained symmetry, unlike LSD and treePL that produced strong left-leaning distributions with modes >–50% underestimates. The underestimation of ΔTEs was much lower for RelTime than treePL and LSD (fig. 2*C*). Therefore, RelTime dealt with rate heterogeneity better than the other two for the data sets analyzed here.

#### Bias in the Estimates of Node Times

**Fig. 3.**
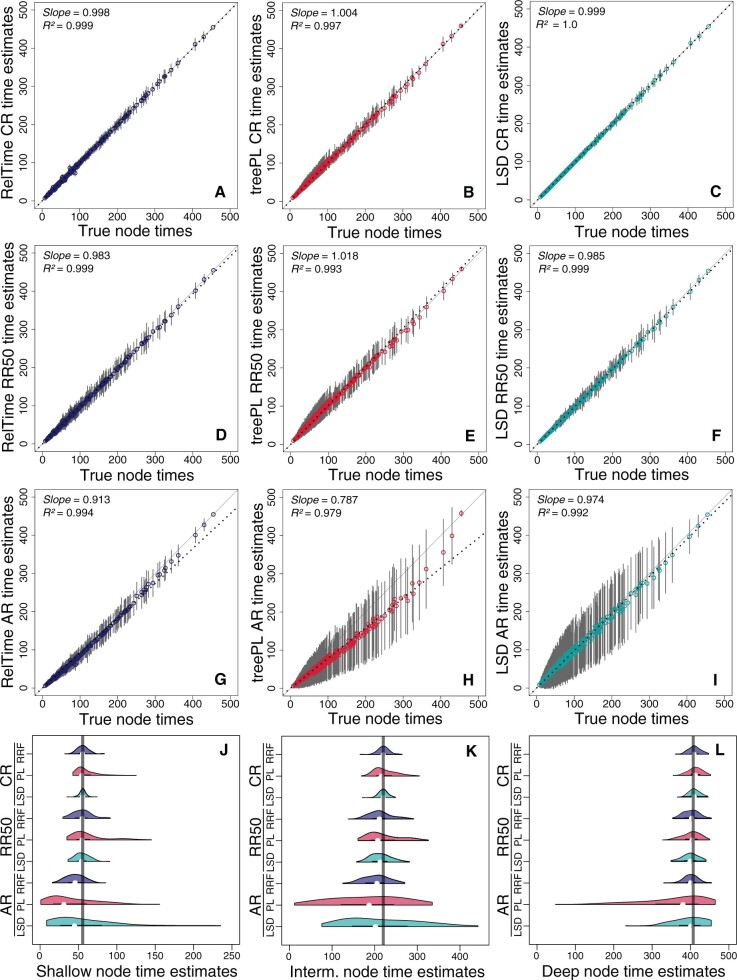
Comparison of time estimates obtained by using RelTime, treePL, and LSD with true node times for CR (*A–C*), RR50 (*D–F*), and AR (*G–I*) data sets. Each data point represents the average of time estimates from 100 simulations (± SD—gray bars). The slope and coefficient of determination (*R*^2^) for the linear regression through the origin are shown. The black dotted line represents the best-fit linear regression through the origin. The solid gray line represents equality between estimates. Distributions of time estimates from three nodes located in different regions in the phylogeny for CR, RR50, and AR data sets: shallow node (*J*), intermediate node (*K*), and deep node (*L*). Comparison is of RelTime (blue curve), treePL (red curve), and LSD (green curve) performance. The gray vertical line represents the true node time. Each distribution of node TEs is computed from 100 simulations.

For each node, the average of estimated times across 100 replicate data sets was used to assess bias. The slope of average node time estimates from the three methods was very close to one when regressed against the true node times for the CR data sets (fig. 3*A*–*C*). For RR50 data sets, the slope was close to one for all three methods (fig. 3*D*–*F*), but treePL estimates displayed a larger dispersion (fig. 3*E*). For AR data sets, RelTime estimates showed a slightly curvilinear relationship due to underestimation of intermediate divergence times (fig. 3*G*). These underestimates were likely the reason for the shift in ΔTEs distribution seen in figure 2*C*, which corresponds to a ∼15% underestimation. For the same data sets (AR), the relationship of time estimates from treePL was much more curvilinear, causing a large bias (fig. 3*H*). The dispersion of treePL time estimates was also much larger than RelTime (compare fig. 3*G* and *H*). LSD time estimates did not show a curvilinear relationship (fig. 3*I*) but a rather large dispersion. This corresponds to the off-center trend observed in figure 2*C*.

We examined the distribution of inferred times from 100 replicate data sets for randomly selected deep, intermediate, and shallow nodes to assess time estimates’ bias in different phylogeny regions (fig. 3*J*–*L*). We found that the dispersion and bias of treePL time estimates for CR and RR50 were larger than RelTime and LSD at all node depths (fig. 3*J*–*L*). For AR phylogenies, both treePL and LSD generated a much larger dispersion than RelTime throughout the phylogeny (fig. 3*J*–*L*).

#### Coverage Probabilities of Confidence Intervals

**Fig. 4.**
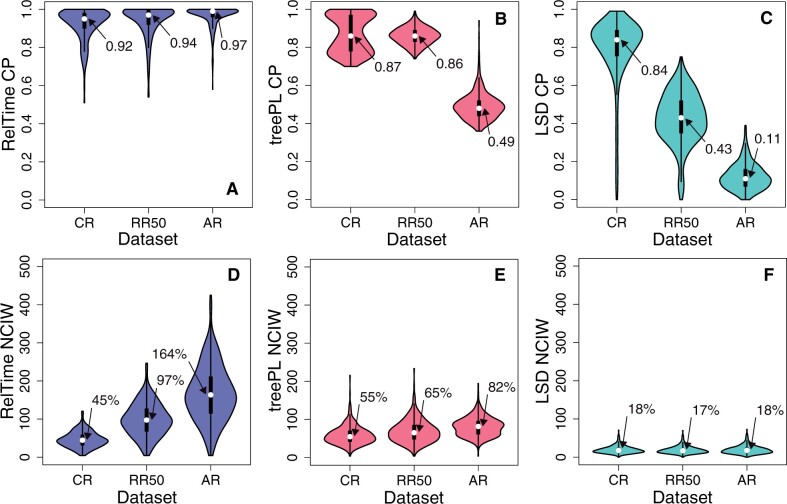
Distribution of coverage probabilities (CP; *A–C*) and mean NCIWs (CI width/true-time × 100; *D–F*) for CR, RR50, and AR data sets. RelTime (blue), treePL (red), and LSD (green) performances are compared. CP and NCIW for each scenario are calculated using the results of 100 simulated data sets. The white dots mark median values.

The accuracy of CIs was measured by coverage probabilities (CP). CP is the proportion of 100 data sets containing the actual node times in the CIs generated by the given method (RelTime, treePL, or LSD). The median CP of RelTime was greater than 0.9 for CR, RR50, and AR data sets (fig. 4*A*), meaning that the estimated CIs contained true time for more than 90% of the data sets in all three cases. In contrast, CPs were much lower for treePL and LSD. For CR data sets, the treePL and LSD CPs were 0.87 and 0.84, respectively (fig. 4*B* and *C*). The performance for RR50 data sets was worse for LSD (0.43, fig. 4*C*) and remained low for treePL (0.86, fig. 4*B*). The performance of CIs for AR data sets was poor for treePL (0.49, fig. 4*B*) and LSD (0.11, fig. 4*C*). These results are partly due to the biased time estimates produced by treePL and LSD (fig. 3*H*–*J*). They are also due to overly narrow CIs of estimated times. We calculated normalized CI widths (NCIW) for node times as CI width divided by true time and then multiplied by 100. In the CR case, the relative widths of CIs were similar for RelTime and treePL, with a median NCIW of 45% and 55%, respectively (fig. 4*D* and *E*). For the same CR data sets, the median NCIW for LSD was much narrower (18%, fig. 4*F*), which resulted in lower CPs.

The difference becomes much greater for phylogenies with extensive rate variation, as RelTime CIs were wider than treePL and LSD. The median NCIW of RelTime estimates was 97% for RR50 and 164% for AR cases (fig. 4*D*). For treePL, the median NCIW was substantially lower than RelTime; 65% and 82% for RR50 and AR case, respectively (fig. 4*E*). For LSD, the median NCIW was very small (∼18%, fig. 4*F*). We note that LSD did not generate CI for all the nodes in the phylogeny because the timetree contained many multifurcations at which the NCIW was equal to zero. Therefore, the lower coverage probability for treePL and LSD was due to narrower CIs and more biased time estimates (fig. 3*B*, *C*, *E*, *F*, *H*, and *I*).

### Impact of Significant Lineage-Specific Rate Shifts on Time Estimates

The model tree in figure 1*E* was used to simulate major lineage-specific rate shifts. Figure 1*F* and *G* shows the impact of rate shifts in which some rates increased three times (3× data sets) or six times (6× data sets). That is, some lineages evolved convergently faster than others in the tree, which has been shown to impact divergence time estimates from Bayesian methods (Beaulieu et al. 2015). We quantified the accuracy, bias, and CPs of time estimates produced by RelTime, treePL, and LSD for these data sets.

#### Accuracy of Individual Node Times

**Fig. 5.**
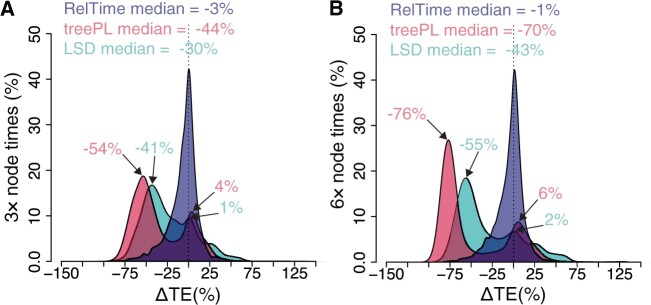
Distributions of the differences between estimated and true node times (ΔTEs) for (*A*) 3× and (*B*) 6× data sets for times inferred by RelTime (blue curve), treePL (red curve), and LSD (green curve). The median of each distribution is shown. Modes of treePL and LSD bimodal distributions are indicated by an arrow. The standard deviations for RelTime, treePL, and LSD, respectively, are as follows in panel (*A*) 17, 31, 30 and (*B*) 17, 40, 36.

We compared the distributions of ΔTEs produced by RelTime, TreePL, and LSD (fig. 5). For RelTime, there is no significant difference in time estimates from 3× and 6× data sets. Both RelTime distributions were centered around zero (fig. 5*A* and *B*, blue curve). However, treePL and LSD ΔTEs for both 3×and 6× data sets deviated significantly from zero. They showed a much larger dispersion, with 6× data sets displaying even larger bias and dispersion. The treePL and LSD ΔTE distributions were bimodal and tended to underestimate times (fig. 5*A* and *B*, red and green curves). Therefore, the median ΔTEs were considerably higher for treePL (–44% and –70% for 3× and 6× data sets, respectively) and LSD (–30% for 3× and –43% for 6×) as compared with RelTime (–3% for 3× and –1% for 6× data sets).

#### Bias in the Estimates of Node Times

**Fig. 6.**
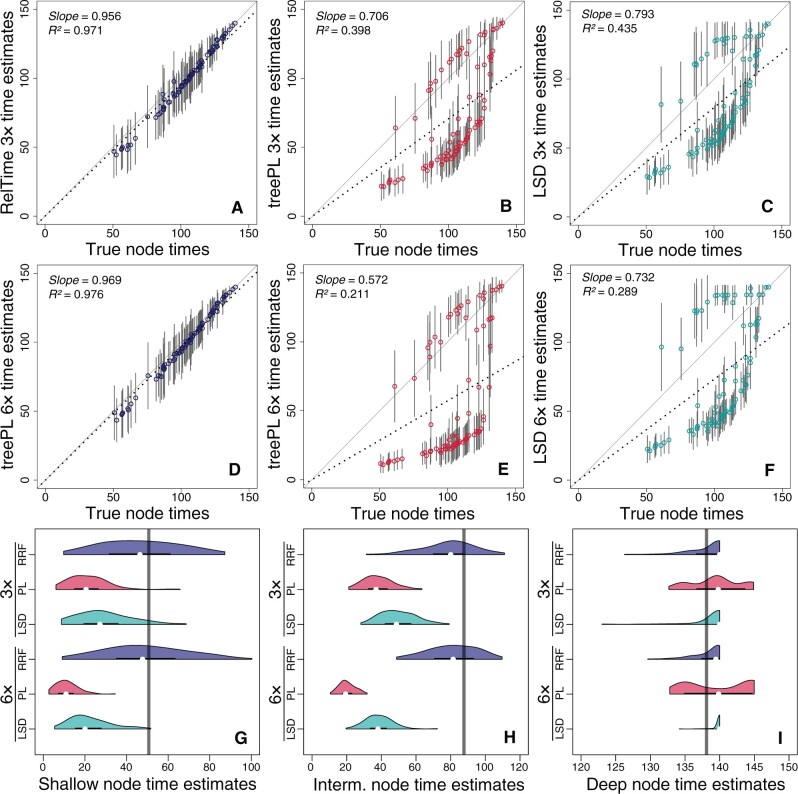
Comparison of time estimates obtained using RelTime, treePL, and LSD with true node times for 3× (*A–C*) and 6× (*D–F*) data sets. Each data point represents the average of time estimates from 100 simulations (± SD—gray bars). The slope and coefficient of determination (*R*^2^) for the linear regression through the origin are shown. The black dotted line represents the best-fit linear regression through the origin. The solid gray line represents the true node time. Distributions of time estimates from three nodes located in different regions in the phylogeny for 3× and 6× data sets: shallow node (*G*), intermediate node (*H*), and deep node (*I*). Comparisons are of RelTime (blue curve), treePL (red curve), and LSD (green curve) performance. The gray vertical line represents the true node time. Each distribution of node TEs is computed from 100 simulations.

We averaged absolute times across 100 data sets for each node and regressed them against the true times. In both 3× and 6× cases, RelTime time estimates were slightly biased because the slopes of average node time estimates were somewhat smaller than one (fig. 6*A* and *D*). In contrast, treePL (fig. 6*B* and *E*) and LSD estimates (fig. 6*C* and *F*) were more biased. For treePL and LSD estimates, the slope was much lower than one for 3× data sets (0.71 and 0.79, respectively, fig. 6*B* and *C*) and even lower for 6× data sets (0.57 and 0.73, respectively, fig. 6*E* and *F*). The large difference in time estimates between 3× and 6× data sets indicates that treePL and LSD are not robust to significant lineage-specific rate shifts. Interestingly, LSD and treePL estimates also showed two trends (fig. 6*B*, *C*, *E*, and *F*), corresponding to the bimodal pattern in ΔTEs (fig. 5).

The distribution of time estimates from 100 replicate data sets for individual shallow, intermediate, and deep nodes from RelTime, treePL, and LSD are shown in figure 6*G*–*I*. RelTime time estimates displayed a large dispersion for shallow and intermediate nodes, but estimates were centered around the true node time (fig. 6*G* and *H*). For the same nodes, treePL and LSD displayed lower dispersions but highly biased distributions far from the true node times (fig. 6*G* and *H*). For deep nodes, all three methods produced times with distributions centered close to the true node time (fig. 6*I*).

#### Coverage Probabilities of Confidence Intervals

**Fig. 7.**
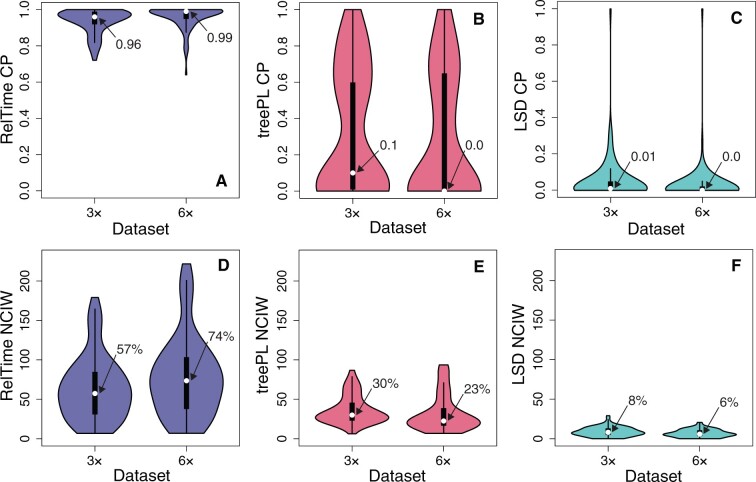
Distribution of coverage probabilities (CP; *A–C*) and mean NCIWs (CI width/true-time × 100; *D–F*) for 3× and 6× data sets. RelTime (blue), treePL (red), and LSD (green) performances are compared. CP and NCIW for each scenario are calculated using the results of 100 simulated data sets. The white dots represent median values.

For both 3× and 6× data sets, RelTime produced excellent CPs. The median CP was 96% and 99% for 3× and 6×, respectively (fig. 7*A*). This was not the case for treePL and LSD estimates; CIs contained the actual node times in a very small proportion of nodes, as the median CP was less than 10% for 3× and 0% for 6× data sets (fig. 7*B* and *C*). This is due to the biased estimates and narrow CIs produced by treePL and LSD. For treePL, the median NCIW were similar for 3× (30%) and 6× (23%) data sets (fig. 7*E*). The performance of LSD was even worse (6–8%; fig. 7*F*). As noted earlier, LSD did not generate CIs for many node times involved in multifurcations.

### A Comparison of Branch Rates

**Fig. 8.**
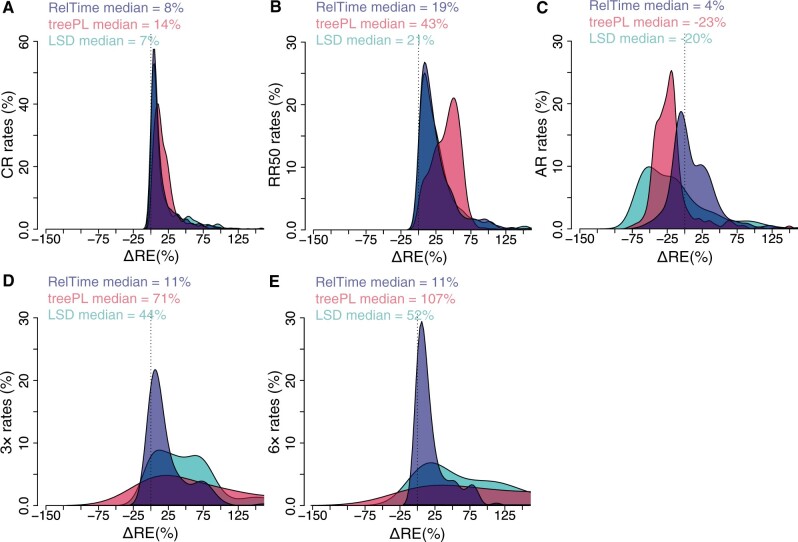
Distributions of ΔREs (estimated rate—true rate/true rate × 100) from individual timetrees from CR, RR50, AR (*A–C*) 3× and 6× (*D* and *E*) data sets for rates inferred by RelTime (blue curve), treePL (red curve), and LSD (green curve). The median of each distribution is shown. The difference between the estimated and true times was computed for every node in the inferred timetrees. The difference was divided by the true rate and multiplied by 100 to generate percent time error (ΔRE). In panels *A* and *B* the distributions of ΔREs for RelTime and LSD are overlapped.

We also examined the accuracy of branch rate estimates from RelTime, treePL, and LSD by comparing the distributions of the differences between the estimated and true branch rates (ΔREs) for a randomly selected phylogeny from each simulation scenario (CR, RR50, AR, 3×, and 6×). The branch rate estimates were computed as the maximum likelihood (ML) branch lengths divided by branch length in time units (ancestor–descendent node times). For CR phylogenies, ΔREs for three methods had a similar distribution that was slightly off-center with a median overestimate of <14% (fig. 8*A*). For the phylogenies in which evolutionary rates were variable (RR50, AR, 3× and 6×), the ΔREs for RelTime showed similar patterns but with larger dispersions. In contrast, ΔREs for treePL and LSD deviated much more from zero. They showed a large dispersion for variable rate phylogenies (fig. 8*B*–*E*). Overall, RelTime rates were more accurate, which is reasonable because node times were estimated more accurately.

## Discussion

We have reported the relative performance of the three methods investigated (RelTime, treePL, and LSD), which depends on the conditions used for simulating sequence data sets. All three methods perform well in estimating node times when evolutionary rates are constant or vary independently among branches. But major differences exist for phylogenies with autocorrelated rates for which RelTime performed better. This result will make RelTime more appealing for analyzing empirical data sets because extensive autocorrelation of molecular rates has been reported in diverse taxonomic groups across the tree of life (Tao et al. 2019). RelTime also did better for some phylogenies in which rates shifted convergently among lineages.

RelTime also produced CIs with expected coverage probabilities, that is, close to 95% success for 95% CIs. In contrast, LSD and treePL yielded CIs with very low CP when rates vary across the phylogeny, making them unsuitable for biological hypothesis testing. The treePL CIs are overly narrow CIs because they were produced using the bootstrap approach. The bootstrap approach only accounts for the errors associated with branch lengths estimation (Thorne and Kishino 2005; Yang 2014). It fails to consider branch rate variation in estimating CIs, which causes CI widths for time estimates from the analysis of CR data sets to be similar to those with extensive rate variation (RR50 and AR) (fig. 4*D*–*F*). The resampling of branch lengths to compute CIs in LSD also does not incorporate the variance due to branch rate variation, which results in narrow CIs for CR, RR50, and AR phylogenies (fig. 4*D*–*F*).

In contrast, RelTime uses analytical equations to properly incorporate the errors associated with branch lengths and the rate variation in CI calculation. Therefore, CIs produced by RelTime are much wider for RR50 and AR data sets than those in the CR case (fig. 4*D*–*F*). These wide intervals are closer to the correct intervals because the expectation of coverage probability of 0.95 is met much better by RelTime CIs. Moreover, the use of the bootstrap approach makes treePL much slower than RelTime and LSD. For example, CI calculations for data sets containing ∼450 taxa required more than 10 days to finish in treePL, whereas LSD and RelTime require only ∼35 and 0.13 s, respectively.

Surprisingly, treePL did not perform well for AR data sets, even though the autocorrelation of branch rates is inherent in the PL framework. This prompted us to analyze some AR data sets in the original PL software, r8s (Sanderson 2003). We found r8s to perform better than treePL for an AR data set in which treePL performed the worst (average ΔTE was 25% for r8s and 75% for treePL for the phylogeny), suggesting that PL implementation in treePL may contribute to its poor performance. In the future, it will be useful to conduct a more extensive comparison of r8s and treePL. But RelTime estimates were significantly better than PL estimates (r8s and treePL) for AR data sets, whereas RelTime and treePL performed similarly for RR50 data sets.

One possible explanation for the difference in the performance of RelTime and PL approaches for AR phylogenies is that AR phylogenies have a much larger local rate variation than RR50 phylogenies (e.g., fig. 1*C* vs *D*). This is because the probability of rate shift was sampled from a lognormal distribution in simulating autocorrelated rates, which results in some clades having much higher rate shifts than their parent clades. When the phylogeny is big, there is a higher chance of having clades with large rate changes. Importantly, unlike the RR50 cases where the rates of branches descending from the branches experienced the extreme rate shift can vary independently and randomly, the rates of the descendant branches will remain extreme because they inherit the large rate change from their ancestor in the AR evolution. When this happens, AR phylogenies show a similar structure as the phylogenies in 3× and 6× rate-shift cases. Using a single, global smoothing parameter to penalize the rate change in phylogenies with large localized rate differences seems unsuitable. In contrast, RelTime does not use a global rate smoothing to control the rate change; rather, it minimizes the rate change between sister lineages and ancestor-descendant lineages at each node in the tree (Tamura et al. 2018). This local rate smoothing seems to give RelTime more flexibility to tolerate large rate shifts, resulting in better rate and time estimates. As for the lower performance of LSD for phylogenies with autocorrelated rates, it is likely because LSD is explicitly designed for phylogenies without correlated rates (To et al. 2016).

In practice, we expect the performance differences among the three rapid methods to decrease when multiple well-constrained calibrations can be applied. In the present study, we used only one root calibration because the focus was on assessing the power of each investigated method independent of the time structure introduced by the calibration priors. It is useful to note that RelTime implementation in MEGA can use probability densities to accommodate calibration uncertainties (Tao, Tamura, Mello, et al. 2020), but treePL and LSD currently only allow calibration boundaries. Ultimately, the use of multiple well-constrained and reliable calibrations is expected to produce better time estimates with higher precision (narrower CIs).

It is important to note that our investigation has been focused on dating a specified phylogeny, thereby assuming no uncertainty in phylogenetic relationships. The addition of this uncertainty may increase the variance of divergence time estimates for some nodes (Ho and Phillips 2009). Therefore, it is a common practice to generate a reliable phylogeny before estimating dates. If the inferred tree is inaccurate, time estimates for many nodes will be meaningless because they would not correspond to actual evolutionary divergence events (Tao, Tamura, and Kumar 2020). In any case, one may apply dating methods to alternative, fully resolved phylogenetic tree topologies and evaluate the robustness of time estimation to uncertainties in the tree topology (dos Reis et al. 2015). Nevertheless, evaluation of the impact of the phylogenetic uncertainty on time estimates is beyond the scope of the current study but will be pursued in future work.

Finally, based on our simulation results, we propose some simple guidelines for rapid molecular dating based on the objective of the molecular dating study. If the primary focus is to estimate divergence times only, one should first test if the evolutionary rates are autocorrelated, for example, by using Tao et al. (2019) method. If so, then RelTime should be considered; otherwise, one may use RelTime, LSD, or treePL. On the other hand, if one needs to estimate divergence times with CIs, then RelTime is preferred for all empirical data analyses (correlated and uncorrelated branch rates) because of RelTime’s high coverage probabilities, accurate times, and much faster speed. It is useful to note that RelTime’s CIs are broader because the analytical approach employed accounts for the variance associated with the branch lengths estimation and the variance due to rate heterogeneity in CI calculation, which is not the case for treePL and LSD CIs.

Moreover, several recent studies using empirical (Mello et al. 2017; Battistuzzi et al. 2018; Tao, Tamura, Mello, et al. 2020) and simulated data (Barba-Montoya et al. 2020; Mello et al. 2021) have reported RelTime to perform as well as Bayesian methods for dating phylogenies with and without calibrations. Therefore, we find that RelTime can be of general use for dating phylogenies, large and small. Whenever feasible, both RelTime and Bayesian methods should be used, and the results compared because no technique is almighty. The concordance of biological inference from methods developed using different frameworks shows the robustness of results to intrinsic assumptions. Such a test will not impose much additional computational expense because RelTime is orders of magnitude faster than Bayesian methods.

## Materials and Methods

### Computer Simulated Data Sets

#### Simulation of Data Sets under Different Rate Variation Processes

We used data sets previously simulated by Tamura et al. (2012). The model timetree consisted of 446 species derived from the bony-vertebrate clade in the Timetree of Life (Hedges and Kumar 2009) (fig. 1*A*). We chose 100 gene alignments in which the rates were clock-like (CR data sets, fig. 1*B*). We also selected another 100 data sets in which the rates varied independently on each branch by ±50% of the overall rate (RR50 data sets; fig. 1*C*). Another 100 data sets chosen were those in which the rate variation was autocorrelated (AR data sets), such that the rate of a descendant branch was drawn from a lognormal distribution around the mean rate of the ancestral branch. For the 100 AR data sets (fig. 1*D*), autocorrelation parameter ν = 1 was used (Kishino et al. 2001). All data sets were generated using SeqGen (Rambaut and Grassly 1997) under the Hasegawa–Kishino–Yano model (Hasegawa et al. 1985) and heterogeneous sets of evolutionary parameters, including sequence lengths (258 to 9353 sites), evolutionary rates (range 1.35–2.60 substitutions per site per billion years), G + C-content bias (G + C contents range 39–82%), and transition/transversion rate bias (transition/transversion ratio, range 1.9–6.0). For more details, refer to Tamura et al. (2012).

#### Simulation of Data Sets with Significant Lineage-Specific Rate Heterogeneity

We used data sets previously simulated by Beaulieu et al. (2015) using a model timetree of 111 taxa (fig. 1*E*) derived from the analysis of the age of land plant clades. The age of crown angiosperms in the model timetree was fixed to be 140 Ma (fig. 1*E*, closed black dot). Assuming an AR among lineages, Beaulieu et al. (2015) simulated scenarios in which four clades in the phylogeny evolved three times higher rate than the remaining taxa (fig. 1*F*). They also simulated a more extreme six times rate difference (fig. 1*G*). Faster branch-specific molecular rates were simulated by independently drawing rates for the branches of four clades within angiosperms (fig. 1*F* and *G*; orange branches) from a lognormal distribution with a mean of 3× higher and 6× higher than all other branches in the tree. The inferred parametric shape of the lognormal distribution of rates from the analysis of land plants (mean = 5 × 10^−4^, Standard Deviation = 0.75) was used as a baseline for increasing the rate of the four clades. Using the model timetree shown in figure 1*E*, 100 data sets with 3× and 100 data sets with 6× rate acceleration were generated and used to simulate sequence alignments of 1,000 sites using SeqGen (Rambaut and Grassly 1997). A general time reversible model of nucleotide substitution was assumed (Tavaré 1986), applying the inferred parameters from the analysis of land plants. For more details refer to Beaulieu et al. (2015).

### Dating by RelTime (Relative Rate Framework)

All RelTime analyses used MEGA-CC for macOS (Stecher et al. 2020). They were prototyped in MEGA X (Kumar et al. 2012, 2018). We used correct species relationships (figs 1*A* and *E*) to avoid confounding the influence of phylogeny accuracy with the performance in estimating time. For all data sets (CR, RR50, AR, 3×, and 6×), branch lengths were calculated using the ML approach and the correct substitution model in MEGA-CC. This phylogeny was then used to infer node times and CIs. To generate trees with absolute times for comparative purposes, we assumed the correct ingroup root node dates. For CR, RR50, and AR, a uniform distribution U(444.6, 464.6 Ma) was specified for the tree in figure 1*A* (closed black dot). The 3× and 6× timetrees were computed using a root calibration only (rooting ingroup node; fig. 1*E*, closed black dot), specified using a uniform distribution U(135, 145 Ma). Dates for all taxa in the outgroup were excluded because RelTime analysis does not produce estimates in the outgroup (for an explanation, refer to Tamura et al. 2012, 2018).

### Dating by treePL (Penalized Likelihood)

The PL dating analysis was conducted in treePL (Smith and O’Meara 2012). As in RelTime analysis, each ML phylogeny with branch lengths was used as an input for PL dating analysis. The best-fit smoothing parameter was specified empirically for each ML phylogeny using a CV test implemented in treePL (Sanderson 2003; Smith and O’Meara 2012). First, the best optimization parameters for each ML phylogeny were determined by prime command. Then, each of the ML phylogenies was subjected to a CV test under the following parameters: cvstart = 10^6^; cvstop = 10^−12^; cvmultstep = 0.1; and the parameters determined by running the prime command. Finally, every ML phylogeny was subjected to a “thorough” dating analysis using the best-fit smoothing parameter inferred in the CV test.

CR, RR50, and AR timetrees were computed applying one calibration on the ingroup node, specified assigning a uniform distribution U(444.6, 464.6 Ma) and a maximum (correct) age constraint of 527 Ma on the rooting outgroup node to ensure that the height of the inferred timetrees matches the model tree height. To generate CIs for the PL dated nodes, we generated 500 bootstrap replicates for each simulated sequence alignment using the model tree (fig. 1*A*) as a constraint in RAxML (Stamatakis 2014). Each ML bootstrap tree was then individually dated using treePL under the same parameters as the single age estimation analysis described above. Then, the 500 dated bootstrap trees were imported into TreeAnnotator (Bouckaert et al. 2019) to calculate the 95% CI for each node.

The 3× and 6× timetrees were computed applying one calibration on the ingroup node, specified assigning a uniform distribution U(135, 145 Ma) and a maximum (correct) age constraint of 317 Ma on the rooting outgroup node to ensure that the height of the inferred timetrees matches the model tree height. One thousand bootstrap replicates for each simulated sequence alignment using the model tree (fig. 1*E*) as a constraint in RAxML (Stamatakis 2014) were computed to generate CIs for the node dates. Each ML bootstrap tree was then individually dated using treePL under the same parameters as the single age estimation analysis. Then, 95% CI for each node was calculated in TreeAnnotator (Bouckaert et al. 2019) by using the 1,000 dated bootstrap trees.

### Dating by LSD (Least-Squares)

The least-squares dating analysis was conducted in LSD-v0.3beta (To et al. 2016). Each ML phylogeny with branch lengths was used as an input for LSD dating analysis to infer node times and CIs. LSD timetrees were estimated excluding the outgroup, assuming correct root node dates and tip dates were set to zero Ma. For CR, RR50, and AR analyses, the root calibration was fixed to 454 Ma for the tree in figure 1*A* (closed black dot). For 3× and 6× data sets, timetrees were computed using a root calibration only (tree in fig. 1*E*, closed black dot), specified using a point calibration of 140 Ma. CIs for the estimated dates were calculated by resampling the branch lengths of the estimated trees 1,000 times to generate a set of simulated trees for CI inference. This method of CI calculation does not involve bootstrap resampling of sites or ML estimation of branch lengths, so it is considerably faster.

### Measurements of Accuracy, Bias, and Coverage Probabilities

The difference between the estimated and true times was computed for every node in the inferred timetrees. The difference was divided by the true time and multiplied by 100 to generate percent time error (ΔTE). A positive value of ΔTE shows an overestimation, and a negative value underestimates true time. Node time bias is the median of ΔTEs from all 100 data sets in the collection (CR, AR, RR50, 3×, or 6×). The percent error in branch rates (ΔRE) is the difference between estimated and the true branch rates divided by the true branch rates and multiplied by 100. We also computed the distribution of time estimates from 100 replicate data sets for randomly selected deep, intermediate, and shallow nodes in the given phylogeny. Shallow nodes are nodes with two descending tips, deep nodes are immediate descendants of the root, and intermediate nodes are nodes whose ages were ∼50% of the root age. The CP of CI for a node’s time is the proportion of data sets in which the CI of the time estimate of that node contained the true time. We also compared NCIW for node times as CI width/true-time × 100.
